# Low-cost gelatin phantom for ultrasound-guided core-needle biopsy training: a pictorial essay

**DOI:** 10.1007/s40477-026-01185-1

**Published:** 2026-08-18

**Authors:** Camilla Culcasi, Lucia Zanchi, Lorena Quagliozzi, Francesca Moro, Federica Pozzati, Valentina Chiappa, Daniela Fischerova, Antonia Carla Testa, Anna Fagotti, Floriana Mascilini

**Affiliations:** 1 Dipartimento di Scienze Della Salute Della Donna, del Bambino E Di Sanità Pubblica, Fondazione Policlinico Universitario Agostino Gemelli IRCCS, UOC Ginecologia Oncologica, Largo A. Gemelli 8, 00168 Rome, Italy; 2 Division of Gynecologic Oncology, Humanitas San Pio X, Milan, Italy; 3 G-AURIG Gemelli Advanced Ultrasound Research and Innovation Group, Fondazione Policlinico Universitario Agostino Gemelli IRCCS, Rome, Italy; 4 UniCamillus – Saint Camillus International University of Health Sciences, Rome, Italy; 5 Unit of Gynecologic Oncology, Fondazione IRCCS Istituto Nazionale Dei Tumori Di Milano, Milan, Italy; 6 Gynecologic Oncology Centre, Department of Gynecology, Obstetrics and Neonatology, First Faculty of Medicine, Charles University and General University Hospital in Prague, Prague, Czech Republic; 7 Department of Life Sciences and Public Health, Università Cattolica del Sacro Cuore, Rome, Italy; 8 Università Cattolica del Sacro Cuore, Rome, Italy

**Keywords:** Ultrasound-guided biopsy, Tru-cut biopsy, Gelatin phantom, Simulation, Gynecologic oncology, Training

## Abstract

Core-needle (tru-cut) biopsy is increasingly used in gynecologic oncology for tissue acquisition and molecular assessment. Adequate operator training is essential, but hands-on opportunities and affordable pelvic simulation models remain limited. This pictorial essay describes a step-by-step approach for pelvic ultrasound-guided core-needle biopsy using a low-cost gelatin-based phantom adapted from a previously published model. The phantom incorporates target lesions and optional simulated bowel loops to increase procedural complexity. The accompanying images and instructional video demonstrate phantom preparation, probe handling, needle-beam alignment, lesion targeting, and simulated biopsy using transvaginal/transrectal and transabdominal approaches. This reproducible, inexpensive model may facilitate simulation-based training and improve familiarity with biopsy equipment and needle-tip visualization before supervised clinical practice.

**Supplementary Information:**

The online version contains supplementary material available at https://doi.org/10.1007/s40477-026-01185-1.

## Introduction

Ultrasound-guided core-needle biopsy is widely used in gynecologic oncology, particularly in patients with advanced, unresectable or recurrent disease, where histological confirmation supports treatment planning and molecular characterization [1–4]. Adequate operator training is essential, yet access to affordable, procedure-specific simulation models remains limited. This pictorial essay provides a detailed, step-by-step description of pelvic ultrasound-guided core-needle (tru-cut) biopsy using a low-cost gelatin phantom based on a previously described model [5]. The procedure simulates transvaginal/transrectal and transabdominal approaches in accordance with ISUOG and ESGO technical recommendations [6], and the accompanying images and instructional video illustrate key procedural components including equipment handling, continuous needle visualization and needle-beam alignment, showing how the technique can be integrated into a structured and reproducible teaching protocol.

### Phantom preparation

**Fig. 1:**
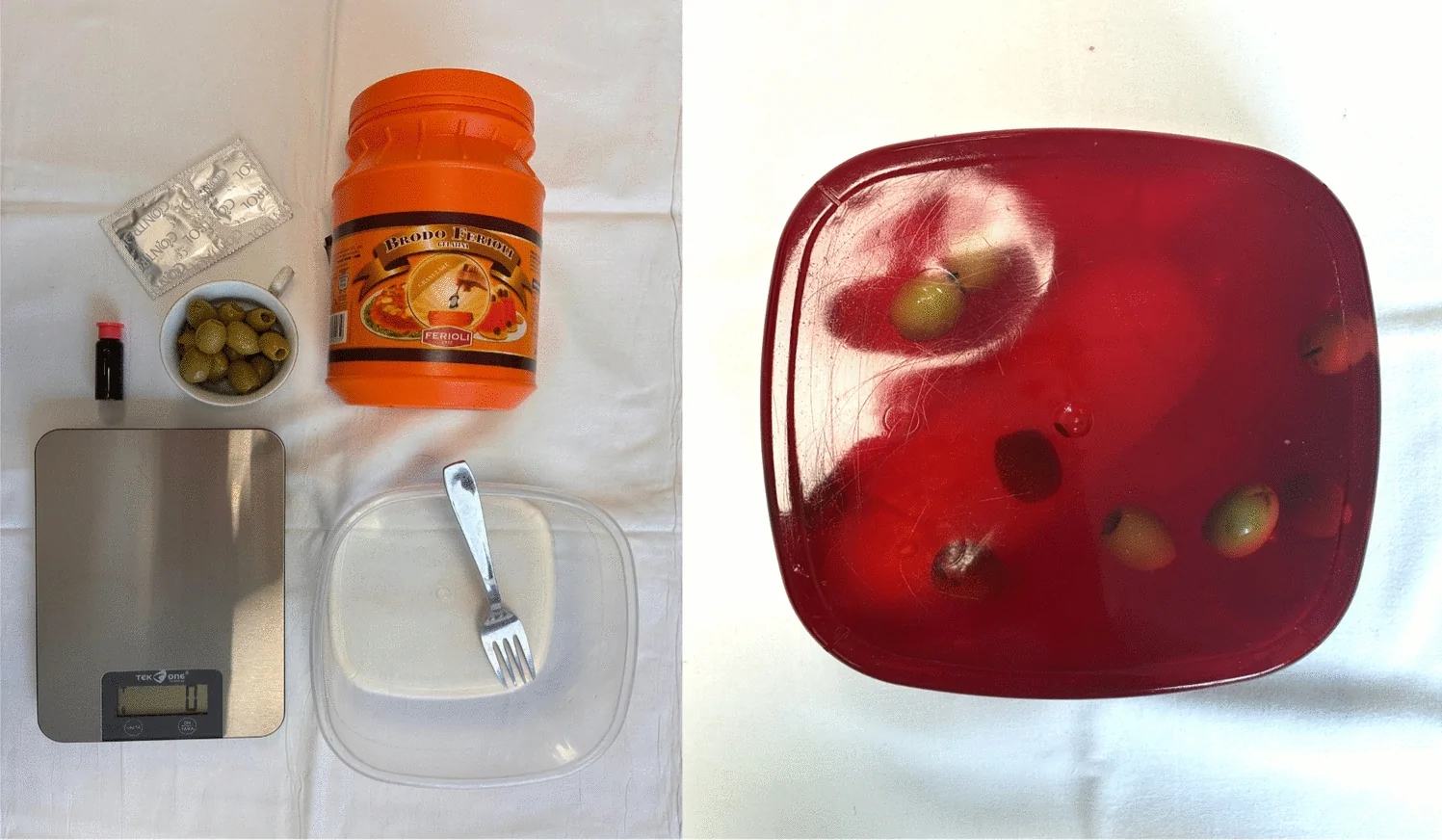
Low-cost gelatin phantom preparation. **a** Materials used for phantom preparation, including food-grade gelatin, kitchen scale, plastic container, fork, food coloring, pitted olives used as target lesions, and fluid-filled condoms used as optional simulated bowel loops. **b** Final appearance of the gelatin phantom after solidification, with embedded target lesions positioned at different depths

A gelatin mixture was prepared using 15 g of powdered gelatin per 100 mL of water, consistent with published low-cost phantom protocols [5, 7]. In our adaptation, water was heated in a microwave oven until near boiling, after which 150 g of gelatin were dissolved in 1 L of hot water with continuous stirring, taking care to avoid air bubbles. The mixture was lightly colored with 5 mL of food coloring and poured into a plastic container. Target lesions consisted of pitted olives, while fluid-filled condoms were incorporated as optional components to simulate bowel loops and increase anatomic complexity. Targets were positioned during the semi-solid phase to prevent sinking. Complete solidification occurred after approximately four to five hours of refrigeration, after which the phantom remained stable at room temperature [5, 7] (Fig. 1). If the phantom solidified before optimal target placement, it was briefly reheated in 10-s microwave intervals to allow repositioning. The preparation protocol is summarized in Table 1.

**Table 1:** Detailed preparation protocol for the 15% w/v gelatin phantom

Step	Description
Ingredients	150 g food gelatin; 1 L hot water; food coloring (optional); pitted olives; fluid-filled condoms to simulate bowel loops
Heat water	Heat water using a microwave (2–3 min intervals) or stovetop until near-boiling
Dissolve gelatin	Add 150 g gelatin to hot water and stir until fully dissolved
Add food coloring	Add 5 mL food coloring to increase opacity
Semi-solid stage	Allow mixture to thicken; insert olives/condoms during semi-solid phase to prevent sinking
Refrigeration	Refrigerate for > = 4 h until fully solidified
Adjustment	If needed, reheat briefly (10-s microwave intervals) to reposition targets

### Equipment and procedural set-up

Ultrasound-guided biopsy simulations were performed using a Samsung Hera I10 system equipped with a high-frequency endocavitary probe for the transvaginal/transrectal approach and with a high-frequency linear probe for the transabdominal approach, as an alternative to the convex probe described in another gynecologic training model [8]. Transvaginal biopsies were performed using an automatic biopsy gun (BioPince, Argon Medical Devices) equipped with an 18-gauge, 25-cm core-cut needle, whereas transabdominal biopsies were carried out using a soft-tissue tru-cut semiautomatic device (PRECISA, H.S. Hospital Service S.p.A.) fitted with a 14-gauge, 10-cm needle. All procedures adhered to the technical principles of the ISUOG-ESGO Consensus Statement [6], adapted to the characteristics of the phantom.

### Endocavitary transvaginal/transrectal simulation

**Fig. 2:**
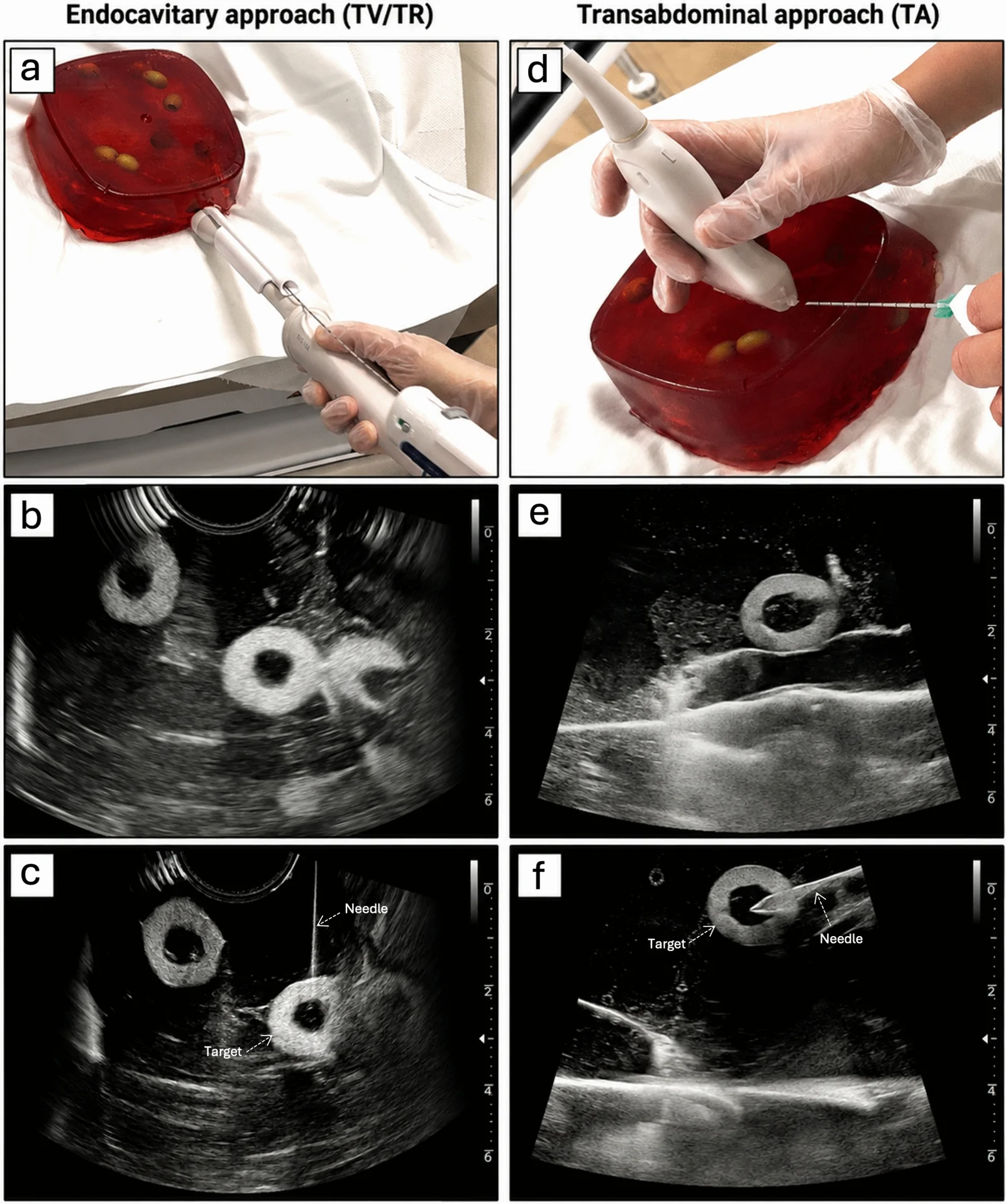
Educational workflow for ultrasound-guided core-needle biopsy simulation using the gelatin phantom. The left column shows the endocavitary transvaginal/transrectal approach, and the right column shows the transabdominal approach. **a** Endocavitary probe fitted with a dedicated needle guide and positioned at the gelatin phantom. **b** Ultrasound visualization of the olive-based target lesions using the endocavitary probe. **c** In-plane needle visualization and target engagement during endocavitary biopsy simulation. **d** Freehand transabdominal set-up using a linear probe and tru-cut needle. **e** Ultrasound visualization of the target lesion during transabdominal simulation. **f** In-plane needle tracking and target engagement during transabdominal biopsy simulation. The complete dynamic sequence is shown in Online Resource 1

For the transvaginal/transrectal simulation, the endocavitary probe was fitted with a dedicated needle guide, ensuring a stable in-plane trajectory and continuous needle visualization. This configuration allowed controlled needle-beam alignment and precise engagement of the olive-based lesions. The endocavitary set-up, target visualization and needle engagement are illustrated in Fig. 2a-c and in Online Resource 1.

### Transabdominal simulation

Transabdominal biopsy was performed freehand using a linear probe; the needle was inserted along the longitudinal axis of the probe, just beneath the ultrasound beam, maintaining constant visualization of the needle tip during advancement. When present, the fluid-filled condom structures representing bowel loops were avoided during trajectory planning. The transabdominal freehand set-up, target visualization and in-plane needle tracking are shown in Fig. 2d-f and in Online Resource 1.

### Educational workflow and role of the video

The educational workflow for using the gelatin phantom can be designed to reflect key principles of ultrasound-guided biopsy training. An expert operator would first demonstrate probe positioning, needle-beam alignment and trajectory planning, providing trainees with a standardized and structured overview of the technique. In accordance with simulation-based learning models, which recommend an initial supervised exposure followed by progressive hands-on practice [8], trainees would first learn the activation mechanism of the automated biopsy device, then plan the needle trajectory before advancing and firing the device within the phantom, targeting the olive-based lesions while avoiding the simulated bowel loops.

A preliminary pilot study of a simulation model for ultrasound-guided core-needle biopsy training in gynaecology has supported the feasibility of this educational approach [9]. The present pictorial essay complements this preliminary evidence by providing a practical, image- and video-supported demonstration of phantom preparation, probe positioning, needle-beam alignment and procedural execution. The accompanying instructional video documents the dynamic sequence of biopsy simulation, complementing the static images and supporting reproducibility of the training protocol.

### Practical performance and limitations

In this educational simulation, active preparation required less than 15 min, with a total material cost below five euros per model. The simulated transvaginal/transrectal and transabdominal approaches allowed stable probe positioning and continuous in-plane needle visualization, with reliable sampling of the embedded lesions. Needle advancement and trajectory planning were reproduced during the simulation, and retrieval of core-like target material was consistently demonstrated. The phantom provided sufficient echogenic contrast and tactile resistance for repeated practice, without relevant structural degradation during the training session. However, the model does not reproduce all clinical conditions, including patient anatomy, respiratory movement, tissue vascularity, bleeding, pain or procedure-related safety constraints. It should therefore be considered a complementary tool for structured training before supervised clinical practice rather than a substitute for clinical experience.

## Conclusion

This pictorial essay provides a clear, reproducible demonstration of how to perform pelvic ultrasound-guided core-needle biopsy using a low-cost gelatin phantom based on a previously described model. The simulation reproduces selected key technical components of both transvaginal/transrectal and transabdominal biopsy, and the accompanying video offers a standardized visual guide that enhances teaching and facilitates adoption. This procedural framework may support structured training for operators with advanced ultrasound expertise, promoting consistent acquisition of biopsy-related skills within competency-based training.

## Supplementary Information

Below is the link to the electronic supplementary material.

Supplementary file1 Instructional video of ultrasound-guided biopsy simulation on the gelatin phantom. The video demonstrates the main steps of the educational workflow, including phantom preparation, target visualization, probe positioning, needle-beam alignment, trajectory planning, and simulated core-needle biopsy using both endocavitary transvaginal/transrectal and transabdominal approaches (MP4 207672 KB)

## Data Availability

All relevant information is included in the article and supplementary material.
